# NORTH CAROLINA NURSING HOME AND PUBLIC HEALTH RESILIENCE DURING 2021 OF THE COVID-19 PANDEMIC

**DOI:** 10.1093/geroni/igac059.1561

**Published:** 2022-12-20

**Authors:** Sandi Lane, Trent Spaulding, Adam Hege, Maggie Sugg, Lakshmi Iyer

**Affiliations:** Appalachian State University, Boone, North Carolina, United States; Appalachian State University, Boone, North Carolina, United States; Appalachian State University, Boone, North Carolina, United States; Appalachian State University, Boone, North Carolina, United States; Appalachian State University, Boone, North Carolina, United States

## Abstract

Since the onset of the COVID-19 pandemic many healthcare organizations have operated in a climate of uncertainty. Building resilience is one method used to survive and thrive during periods of uncertainty. Measuring an organization’s resilience (ability to prepare for, respond and adapt during time of change) allows the organization to identify its vulnerabilities and set priorities to avert negative outcomes during an untoward event. Through collaboration with four long-term care professional associations, the Benchmark Resilience Tool (BRT-13) resilience survey was disseminated to North Carolina long-term care leaders in April 2021. The BRT-13 survey was also sent to North Carolina public health officials via email during the same timeframe. The BRT-13 contains 13 resilience (RES) items divided into two factors of adaptive capacity (AC) and planning (PL) on a five-point Likert scale of strongly disagree (1) to strongly agree (5). Organizational factors surveyed included type of facility, rural-urban classification area designation, ownership type, level of debt, level of profitability, and employee satisfaction. A total of 142 completed surveys were received, 101 (71%) from long-term care leaders and 41 (28.9%) from Public Health officials. Overall average resilience scores ranged from 3.96 for public health respondents to 4.46 for continuing care retirement communities (CCRC) respondents. Analysis of Variance (ANOVA) was employed to compare the three factors (AC, PL, and RES) to the organizational factors. Resilience was significantly associated with one factor, employee satisfaction. Our findings indicate that organizations can build resilience through processes that contribute to staff satisfaction.

